# Mapping suitable habitats for globally endangered raptors in Kenya: Integrating climate factors and conservation planning

**DOI:** 10.1002/ece3.10443

**Published:** 2023-08-31

**Authors:** Peggy Mutheu Ngila, David Odhiambo Chiawo, Margaret Awuor Owuor, Vivian Oliver Wasonga, Jane Wangui Mugo

**Affiliations:** ^1^ Department of Land Resource Management and Agricultural Technology University of Nairobi Nairobi Kenya; ^2^ Center for Biodiversity Information Development (BID‐C) Strathmore University Nairobi Kenya; ^3^ School of Environment Water and Natural Resources South Eastern Kenya University Kitui Kenya; ^4^ Wyss Academy for Nature University of Bern Bern Switzerland; ^5^ Department of Earth and Climate Science University of Nairobi Nairobi Kenya

**Keywords:** raptor conservation, raptors, raptors‐habitat suitability, species distribution modeling

## Abstract

Raptors face global threats like electrocution, collisions, and habitat fragmentation. Many species remain understudied, and their distribution patterns are unknown. Understanding their current and future distribution is crucial for conservation. Protecting these top predators requires knowledge of their spatial distribution and environmental influences. This study addresses knowledge gaps in raptor habitats and distributions in Kenya, considering current and future climate changes. Using species distribution models and occurrence data from the Global Biodiversity Information Facility, we evaluated suitable habitats for four endangered Kenyan raptor species: Martial eagle, Secretarybird, Bateleur, and Steppe Eagle. We assessed the impact of climatic predictors on their distribution, considering two climate change scenarios for 2020–2040. Our findings reveal that raptor distribution in Kenya is predominantly concentrated in the southwestern region, extending into the central region of the country. The most significant predictors of raptor species distribution varied for each species, with Steppe eagle and Secretarybird being highly influenced by precipitation during the warmest quarter, Martial eagle being influenced by mean temperature during the driest quarter, and Bateleur being primarily influenced by precipitation during the coldest quarter. When projecting our model into the climate change scenarios for 2020–2040, all species except the Bateleur exhibited a negative range shift. The results of our study suggest that climate change may have adverse impacts on the raptor species examined. In light of these findings, we recommend implementing targeted monitoring and conducting surveys in accordance with our current model predictions. Specifically, our focus should be on monitoring areas that exhibit the highest climate suitability, as these areas are likely to undergo significant shifts in the near future. By conducting regular monitoring and engaging in further research, we can enhance our understanding of these raptor species and gather valuable data to improve the accuracy and reliability of our model predictions.

## Graphical abstract

Spatial patterns and future projections of raptors in Kenya in the face of climate change.

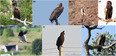

## 1 INTRODUCTION

Globally, 52% of raptor species are in decline, and 18% are classified as threatened with extinction (see McClure et al., 2018). Notably, certain countries such as Indonesia, Tanzania, Sudan, and Kenya harbor the highest number of threatened species (Cruz et al., 2021; McClure et al., 2018). The most prominent causes of raptor population declines are habitat destruction or alteration (Thiollay, 1998; Virani & Watson, 1998) intentional killing (Brochet et al., 2019), intentional and unintentional poisoning (Garbett et al., 2018; Oaks et al., 2004; Ogada et al., 2016) electrocution (Lehman et al., 2007; Mojica et al., 2018), and climate change (Franke, 2017; Iknayan & Beissinger, 2018; Monadjem et al., 2013).

Kenya is home to an impressive array of raptors, with 102 different species documented, and approximately 14% of them facing global threats (Birdlife International, 2021; Ogada et al., 2022). A recent study focusing on raptors in Kenya has uncovered distressing historical trends and recent assessments indicating a staggering alarming decline of over 50% in population numbers over a 40‐year period (Ogada et al., 2022, Figure 1). Kenya's climate variability, characterized by recurrent droughts and floods, has exerted adverse effects on the environment (Kogo et al., 2021). Consequently, it becomes crucial to identify the present and future distribution of these species to formulate effective management strategies and evaluate their conservation status within this rapidly changing environment (Lawler et al., 2011; Miller, 2010).

**FIGURE 1:**
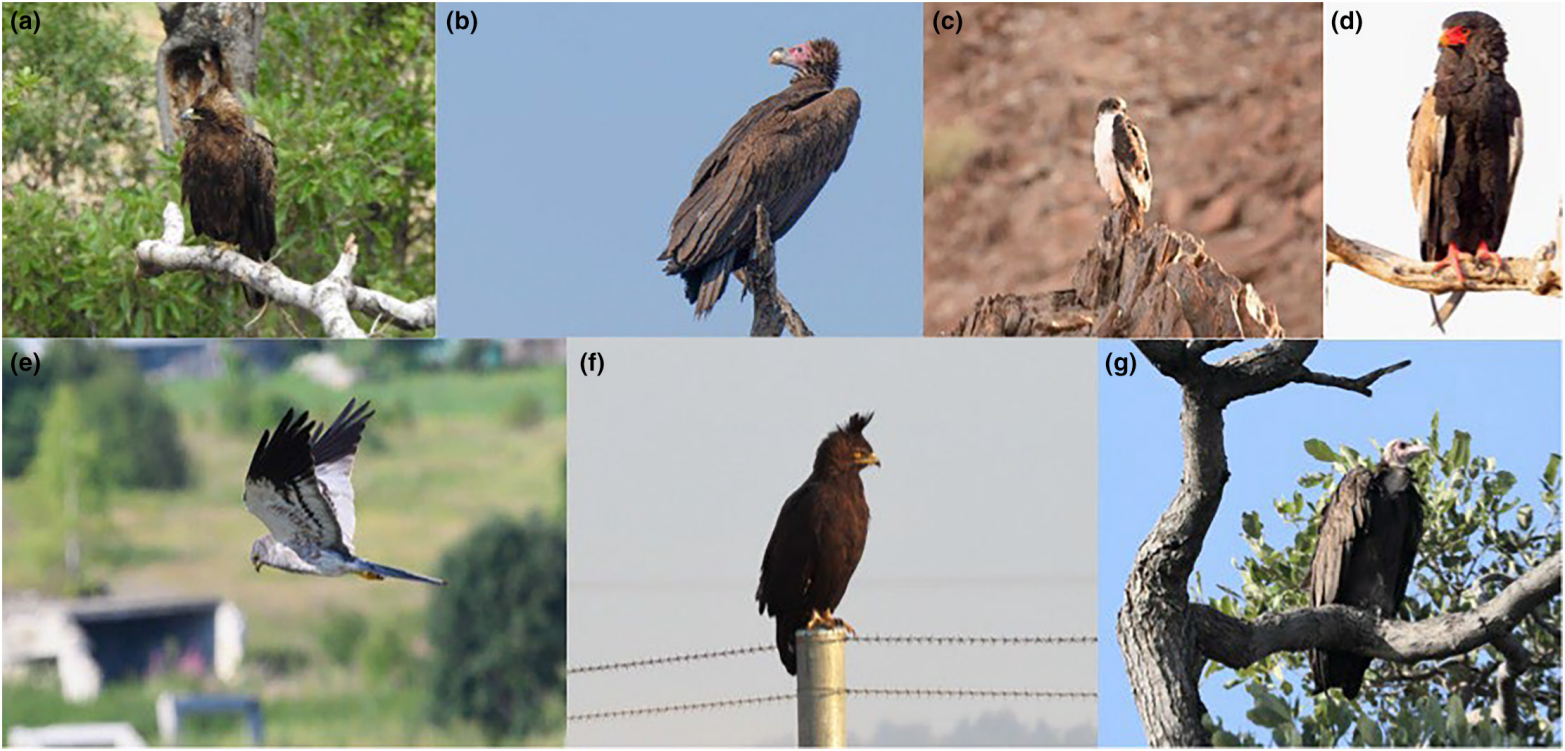
Examples of raptor species in Kenya and their conservation status. Some of these species have experienced declines at the national level, which have not yet been recognized by the IUCN. (a) Wahlberg's eagle (*Hieraaetus Wahlberg*) – (© Lynne Correia) http://creativecommons.org/licenses/by‐nc/4.0/ (b) Lappet‐faced vulture (*Torgos tracheliotos*) – (© petermcintyre) http://creativecommons.org/licenses/by‐nc/4.0/ (c) Augur buzzard (*Buteo augur*) – (© Russ Hoverman) http://creativecommons.org/licenses/by‐nc/4.0/ (d) Bateleur (*Terathopius ecaudatus*) – (© petermcintyre) http://creativecommons.org/licenses/by‐nc/4.0/ (e) Montagu's harrier (*Circus pygargus*) – (© mariula92) http://creativecommons.org/licenses/by‐nc/4.0/ (f) Long‐crested eagle (*Lophaetus occipitalis*) – (© moxcalvitiumtorgos) http://creativecommons.org/licenses/by‐nc/4.0/ (g) Hooded vulture (*Necrosyrtes monachus*) – (© Rob Van Epps).

Recent advancements in the understanding of raptors, as presented by Mcclure et al. (2019), have introduced a comprehensive redefinition of these species that surpasses the sole reliance on morphological characteristics. This novel definition takes into consideration phylogeny, morphology, and ecology, with a significant focus on utilizing evolutionary history to reveal shared patterns of common ancestry. According to this updated definition, raptors encompass all species within various orders that trace their origins back to a raptorial land bird lineage, where the majority of species have retained their raptorial lifestyles inherited from a common ancestor. Consequently, this inclusive definition encompasses species found within the orders Accipitriformes (hawks, eagles, kites, old world vultures), Cathartiformes (new world vultures), Strigiformes (owls), and Falconiformes (Falcons and caracaras; Hackett et al., 2008; Prum et al., 2015).

The importance of raptors extends beyond their individual conservation status. These birds have been recognized as potentially valuable surrogate species for the overall preservation of biodiversity (Sergio et al., 2006, 2008). Positioned at the apex of environmental food chains, raptors play a crucial role as custodians, offering early warnings of potential human‐induced impacts on biodiversity in the face of climate change (Burfield, 2008; Donázar et al., 2016). Furthermore, their dynamic habitats and diverse prey make raptors ideal candidates for umbrella species, representing species diversity across the food chain (Burgas et al., 2014; Sergio et al., 2008). It is worth noting that, apart from vultures, raptors enjoy considerable popularity among the general population and have been extensively studied by scientists due to their intrinsic appeal and recognition (Buechley et al., 2019). This popularity positions raptors as flagship species, effectively mobilizing resources and support for the conservation and protection of biodiversity as a whole (Donázar et al., 2016; McGowan et al., 2020). Consequently, raptors serve as valuable and important study systems for investigating the effects of climate change (Donázar et al., 2016).

Raptors' movements and feeding preferences are known to be influenced by various environmental variables, including precipitation, temperature, human influence, and vegetation, as demonstrated in numerous studies (e.g., Smeraldo et al., 2020; Zhang et al., 2019). Understanding the relationships between raptor distribution and these environmental predictors can help determine their ideal niche. Such information is crucial for developing spatially explicit management and conservation measures, especially when combined with climatic and anthropogenic variables (Smeraldo et al., 2020; Zhang et al., 2019).

Accurately determining species occurrence plays a pivotal role in making informed decisions regarding biodiversity conservation policies. It directly impacts various aspects such as nature reserve selection (Cabeza & Moilanen, 2001), biological invasion monitoring (Gormley et al., 2011), identifying vital habitats for endangered species (Brotons et al., 2004) and provides the backbone for most International Union for Conservation of Nature (IUCN) Red List assessments, as Criterion B is based on a species' extent of occurrence (EOO) and area of occupancy (AOO) polygons which are usually calculated on occurrence points.

This type of data is often collected arbitrarily and made accessible through sources like museum records or biodiversity data websites such as the Global Biodiversity Information Facility (GBIF; Sardà‐Palomera et al., 2012), actively searching for new presence locations of endangered and rare species is essential to enhance management and conservation efforts. Understanding their distribution can provide valuable insights (Guisan et al., 2006). Species distribution models (SDMs) are widely used tools that help deduce ecological requirements and predict the geographic distribution of species. They have gained significant importance in various applications, including regional biodiversity assessment, conservation planning, and wildlife management (Elith & Leathwick, 2009).

Bateleur (*Terathopius ecaudatus*) is an Endangered species, as listed in the IUCN red list of species (IUCN, 2020). It is endemic to Africa and smaller parts of Arabia and is characteristically a bird of somewhat open habitats such as savanna with some trees present and open dry woodland (Ferguson‐Lees & Christie, 2001). Over the past three generations, it has experienced significant declines primarily due to deliberate and incidental poisoning, as well as exposure to pesticides and nest disturbances (IUCN, 2020). Similarly, the Secretarybird (*Sagittarius serpentarius*) has been classified as Endangered, with recent evidence indicating severe population declines across its entire range. The main threats to this species include habitat degradation, disturbance, hunting, and capture for the trade. The Steppe eagle (*Aquila nipalensis*) is found in various parts of Africa, Europe, and Asia. While it has faced rapid declines within its European range, recent information suggests that populations outside Europe may also be at greater risk than previously anticipated. Consequently, the Steppe eagle is now classified as Endangered. The Martial eagle (*Polemaetus bellicosus*) is an extant resident bird found in several countries across sub‐Saharan Africa. Its range extends from Senegal and the Gambia in the west to Ethiopia and north‐west Somalia in the east, and south to Namibia, Botswana, and South Africa (Ferguson‐Lees & Christie, 2001). It is listed as Endangered (IUCN, 2020), as it has experienced significant declines over the past three generations. The main factors contributing to its decline include deliberate and incidental poisoning, habitat loss, reduction in available prey, pollution, and collisions with power lines.

Against this background, this study aims to increase understanding of the biogeographical information of four raptor species in Kenya: the Martial eagle, Bateleur, Secretarybird, and Steppe eagle, for use in conservation actions and management. The specific research objectives are as follows: (1) identify the ecological niche and geographic distribution of four raptor species in Kenya; (2) determine the relative importance of climatic variables influencing raptor distribution; and (3) provide recommendations for raptor habitat management and protection in Kenya. The results of this study will significantly contribute to our understanding of raptors' ecological niches and the critical climatic factors that influence their distribution. Ultimately, this knowledge will inform sustainable management efforts to effectively conserve and protect raptor habitats.

## 2 DATA AND METHODS

### 2.1 Study area

Approximately 580,367 km^2^ in size, Kenya is situated between latitudes 5 N and 5 S and longitudes 34 and 42. Kenya's predominant bimodal rainfall and temperature patterns are determined by the Inter‐Tropical Convergence Zone (ITCZ). The country's rainfall patterns are governed by the seasonal variability and intensity of ICTZ due to differences in altitude.

Kenya's topography is diverse, with elevations ranging from 0 to 5197 m above sea level. The dry land mass is typically represented by six agroecological zones: agroalpine (0.1%), high potential (9.3%), medium potential (9.3%), semi‐arid (8.5%), and dry (52.9%). Agro‐pastoralists and pastoralists predominately live in the semi‐arid to very arid zones which make up 80% of the country (Ngila et al., 2017). Kenya has 28 national reserves totaling 18,042 km^2^ (11.7%), 22 national parks totaling 29,357 km^2^ (5.16%), and 160 conservancies totaling 36,300 km^2^ (11.0%). Kenya has 68 important biodiversity areas (IBAs), with 55 of them threatened (Birdlife International, 2022).

### 2.2 Data pre‐processing and cleaning

We obtained a total of 420 occurrence records for the four raptor species (Bateleur – 160 (https://doi.org/10.15468/dl.ufdwfy), Martial eagle – 98 (https://doi.org/10.15468/dl.utpbre), Secretarybird – 102 (https://doi.org/10.15468/dl.8ncc6f), and Steppe eagle – 60 (https://doi.org/10.15468/dl.2v9353)) across Kenya from the Global Biodiversity Information Facility (GBIF) database (www.gbif.org). Occurrence records utilized in this study obtained from the GBIF database aggregates data from various sources, including the reliable “Bird of Atlas Kenya” dataset that undergoes rigorous quality checks (Pomeroy, 1989) and observer programs. To ensure data integrity, we employed the *clean_coordinates* wrapper function from the CoordinateCleaner package (Zizka et al., 2019). This function facilitated the removal of records with zero coordinates, coordinates displaying inconsistencies with country information, outlier coordinates, coordinates associated with biodiversity institutions, coordinates linked to country and province centroids, as well as coordinates falling within urban areas. In addition to geographic cleaning, we imposed criteria to include only records at the species level and specific to the taxon of interest.

In order to address issues of spatial autocorrelation and sampling bias in the occurrence data, a spatial filtering technique was employed to mitigate model over‐fitting (Boria et al., 2014; Radosavljevic & Anderson, 2014). Compared to alternative sampling bias correction methods (Fourcade et al., 2014; Kramer‐Schadt et al., 2013), spatial filtering has demonstrated superior performance by reducing omission errors and enhancing model predictive accuracy (Aryal et al., 2016). To minimize the impact of oversampling in extensively surveyed regions, a spatial filter distance of 40 km was adopted for each species, based on findings from a previous study on falcons (Sutton et al., 2020). The *thin* algorithm function from the R package SpThin (Aiello‐Lammens et al., 2015) was employed to identify and exclude clustered occurrence points, determining the selection criteria for point inclusion.

Subsequently, 351 records remained after the cleaning process, which was used to construct the Species Distribution Models (SDMs). The distribution of records after cleaning among the raptor species was as follows: Bateleur – 140 (20 records removed), Martial eagle – 86 (12 records removed), Secretarybird – 70 (32 records removed), and Steppe eagle – 55 (5 records removed).

### 2.3 Climatic predictors

Nineteen Bioclimatic variables for current distributions were obtained from Worldclim database (version 2.1, Fick & Hijmans, 2017; https://www.worldclim.com/current). In order to focus on changing climatic conditions while keeping other factors constant, we exclusively utilized climatic predictors instead of incorporating topographical or habitat variables (e.g., Sutton et al., 2020). Although various factors can influence species distribution models, reliable projections for future distribution changes can only be constructed based on predictions from future climate models. While we currently lack the ability to precisely predict habitat changes by 2050, a range of global climate change models can be employed to generate predictions based on existing climate constraints. Moreover, on a broader scale, climate is widely recognized as the primary driver of species distributions, making bioclimatic predictors the most suitable variables to employ (Pearson & Dawson, 2003).

It is important to consider that models can be biased by multicollinearity among environmental predictor variables, which may exaggerate the biological significance of correlated variables (Franklin, 2009). We used the “usdm” package in R to carry out a variance inflation factor stepwise procedure to decrease multicollinearity in predictor variables (Naimi et al., 2014). Variables with variance inflation factors >10 were eliminated. As a result, we only kept the 8 best‐fitting predictors (temperature seasonality, annual temperature range, mean temperature of driest quarter, precipitation of wettest quarter, precipitation of driest month, precipitation seasonality, precipitation of warmest quarter, precipitation of driest quarter) based on the four raptors' ecological requirements.

In our study, we employed one specific General Circulation Model (GCM) derived from the Hadley Centre Global Environment Model (HadGEM3) to generate predictions for the distribution patterns of four raptor species in future climate scenarios. The time frame considered for these predictions was 2021–2040. To obtain the necessary climatic data, we sourced information from the WorldClim database (Version 2.1, Fick & Hijmans, 2017). Additionally, we incorporated two distinct Shared Socioeconomic Pathways (SSPs) that offer diverse outlooks on global developments, presenting varying challenges for climate change mitigation and adaptation. These SSPs are constructed based on five narrative scenarios, encompassing sustainable development, regional rivalry, inequality, fossil‐fueled development, and middle‐of‐the‐road development (O'Neill et al., 2017; Riahi et al., 2017).

Within the context of sustainable development pathways, the reference scenario was set as SSP2, also known as the middle‐of‐the‐road scenario. For the development of future climatic scenarios, we specifically selected SSP245 (middle‐of‐the‐road development) and SSP585 (fossil‐fueled development).

### 2.4 Species distribution models

We accounted for the strengths and weaknesses of different SDM approaches, including regression‐based models and machine learning approaches. Three algorithms (GLM, MAXENT, and GBM) were run by applying an ensembling approach using the “biomod2” (version 4.2‐4) package's ensemble forecasting method (Thuiller et al., 2009) found in R. Generalized linear models (GLMs) were readjusted using a binomial link function. On the other hand, GBMs were generated by performing 5000 three‐fold cross‐validation procedures to determine the optimal number of trees to keep and a maximum depth of variable interactions of 7. The default settings and the highest iteration count of 1000 were applied to MAXENT models. We added a background set of 10,000 randomly chosen background points to the study area because our dataset only contained presence data. As in previous research with species distribution modeling, the occurrence dataset was randomly divided into a 30% sample for evaluating the performance of the model and a 70% sample for model calibration (Smeraldo et al., 2020). We performed 60 SDMs in total (three algorithms × five splitting replicates for model evaluation × one repetition × four species).

### 2.5 Model evaluation

The Area under the receiver operating characteristic curve (AUC) was used to assess the models' predictive performance of the models (Hanley & McNeil, 1982) and the True Skill Statistic (TSS; Allouche et al., 2006). The sensitivity, or the percentage of known presences predicted as presences, is plotted on the ROC curve against specificity, or the percentage of pseudo‐absences predicted as absences. These validation techniques are well‐known and perform very well (Breiner et al., 2015; Smeraldo et al., 2018, 2020). Models with an AUC of <0.7 were disqualified. It has been demonstrated that weighing the individual model projections according to their AUC scores is a particularly trustworthy technique (Marmion et al., 2009; Smeraldo et al., 2020). Additionally, the ensemble model's relative importance of the variables was calculated from the “biomod2” package devoted functionality (Jiguet et al., 2010). The ensemble models' projections from the two‐stage sampling mentioned above were averaged to produce the final potential distribution. To evaluate the spatiotemporal habitat dynamics, the ensemble models were projected at roughly 1 km resolution.

For future predictive models, we also calculated an ensemble forecast for current time and the two climatic SSP scenarios mentioned above. For this purpose, we used weighted mean average based on the AUC values. We used the BIOMOD_RangeSize function from the Biomod package to compare the range sizes between the current projection and the two future climatic scenarios.

## 3 RESULTS

### 3.1 Model performance

The ensemble models' performance varied among the species based on true skill statistic (TSS) and area under the curve (AUC) values as shown in Table 1. The Steppe eagle exhibited high accuracy, with TSS sensitivity and specificity values at 90.74% and 78.86%, respectively, and AUC sensitivity and specificity values at 90.74% and 79.43%. The Bateleur achieved reasonable sensitivity of 73.91% for both TSS and AUC, with specificity values of 76.52% (TSS) and 77.75% (AUC). The Martial eagle showed sensitivity values of 82.14% (TSS) and 82.14% (AUC), with specificity at 66.20% (TSS) and 66.96% (AUC). For the Secretarybird, sensitivity values were 95.46% (TSS) and 83.33% (AUC), and specificity values were 62.94% (TSS) and 75.52% (AUC). The models demonstrated varying performance levels, with Steppe eagle and Secretarybird achieving higher accuracy in classifying instances specific to their species.

**TABLE 1:** The table shows the predictive performance of the SDMs as indicated by the AUC and TSS values shown.

Species	TSS	AUC
Steppe eagle	Sensitivity – 90.74 Specificity – 78.86	Sensitivity – 90.74 Specificity – 79.43
Bateleur	Sensitivity – 73.91 Specificity – 76.52	Sensitivity – 73.91 Specificity – 77.75
Martial eagle	Sensitivity – 82.14 Specificity – 66.20	Sensitivity – 82.14 Specificity – 66.96
Secretarybird	Sensitivity – 95.46 Specificity – 62.94	Sensitivity – 83.33 Specificity – 75.52

### 3.2 Current distribution of the four raptor species

**FIGURE 2:**
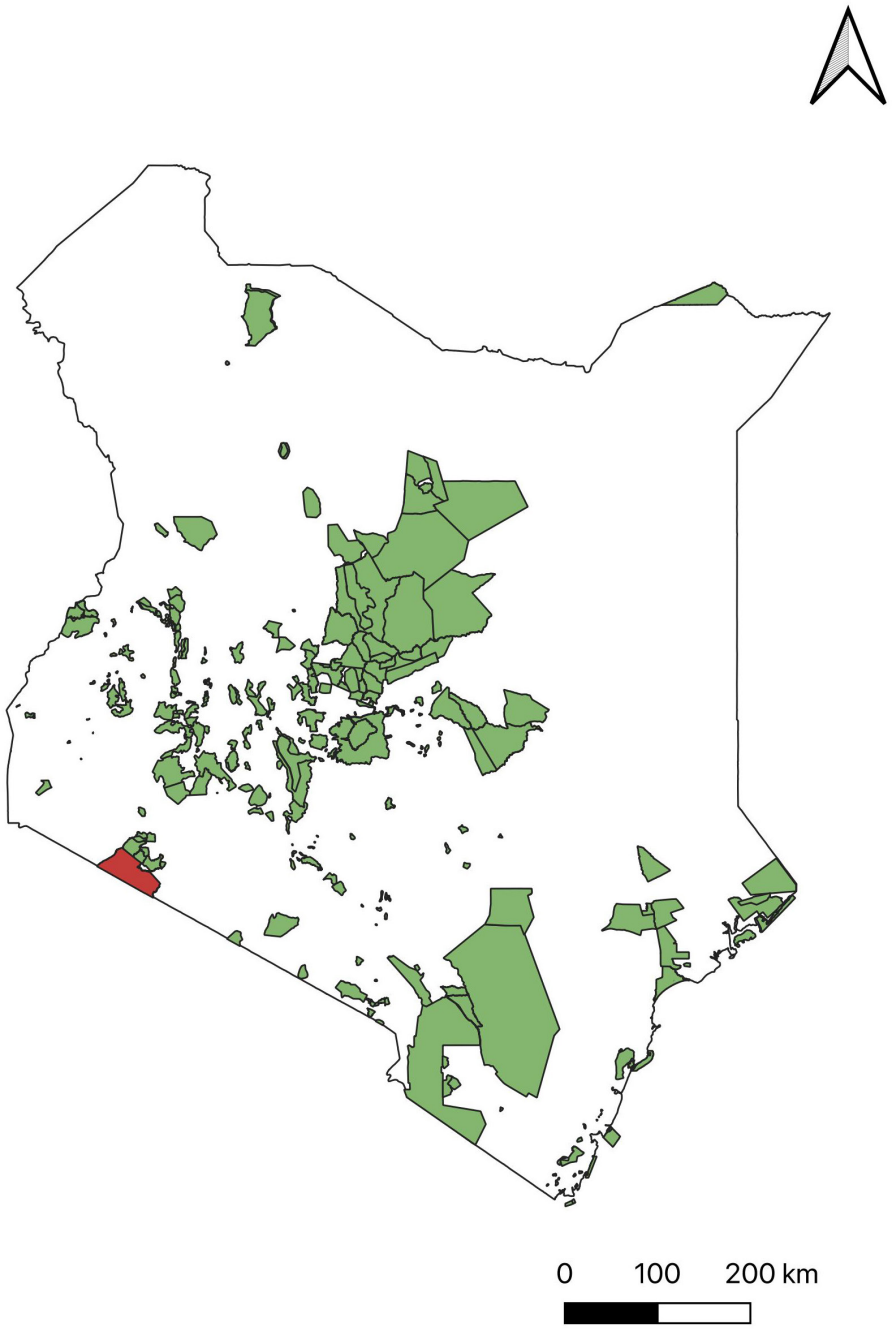
Map showing protected areas in Kenya. Suitability of most raptors species are in the South‐Western part of the country extending towards the central region. Masai Mara game reserve is highlighted in red as most species' suitability is within this region.

**FIGURE 3:**
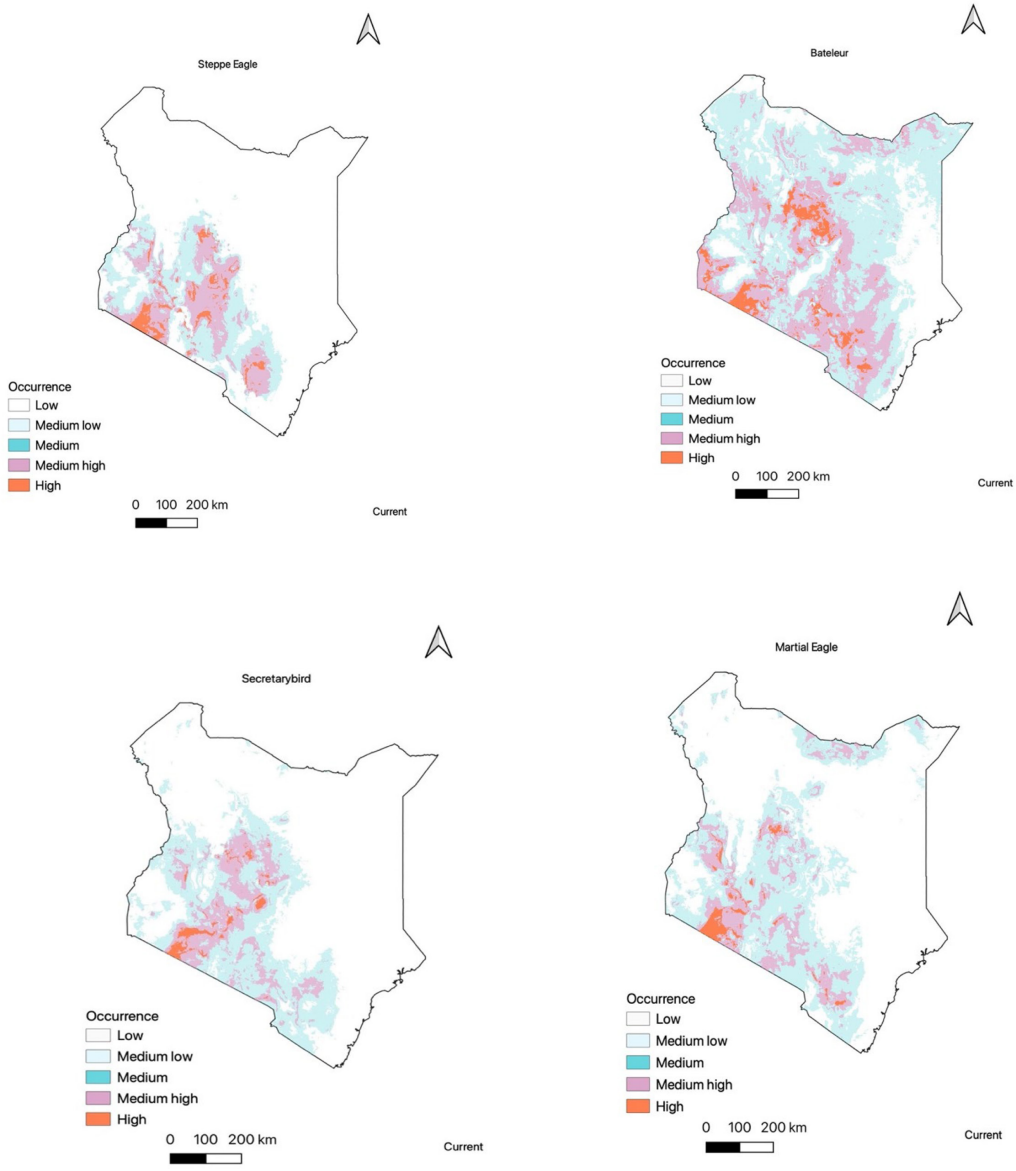
Predicted distribution model of all the four raptor species using current projections. The maps show a continuous logistic prediction with orange areas having the highest climatic suitability. White areas show areas with low climatic suitability from the ensemble model.

In the present study, the utilization of occurrence data has facilitated the creation of a continuous predictive map illustrating the habitat suitability of raptor species in Kenya. The findings indicate that the southwestern and central regions of the country, along with the northern areas for species such as the Bateleur and Martial eagle, exhibit a high prevalence of suitable habitat based on the bioclimatic variables. Particularly, the southwestern region, proximate to Masai Mara National Park (Figure 2), demonstrates the highest level of climatic suitability for all four species, with this favorable habitat extending towards the central parts of Kenya. Notably, the Bateleur species demonstrates the most significant degree of climatic suitability, as it exhibits moderate suitability throughout the majority of the country, with the exception of the eastern region (Figure 3).

### 3.3 Variable importance of climatic variables

**FIGURE 4:**
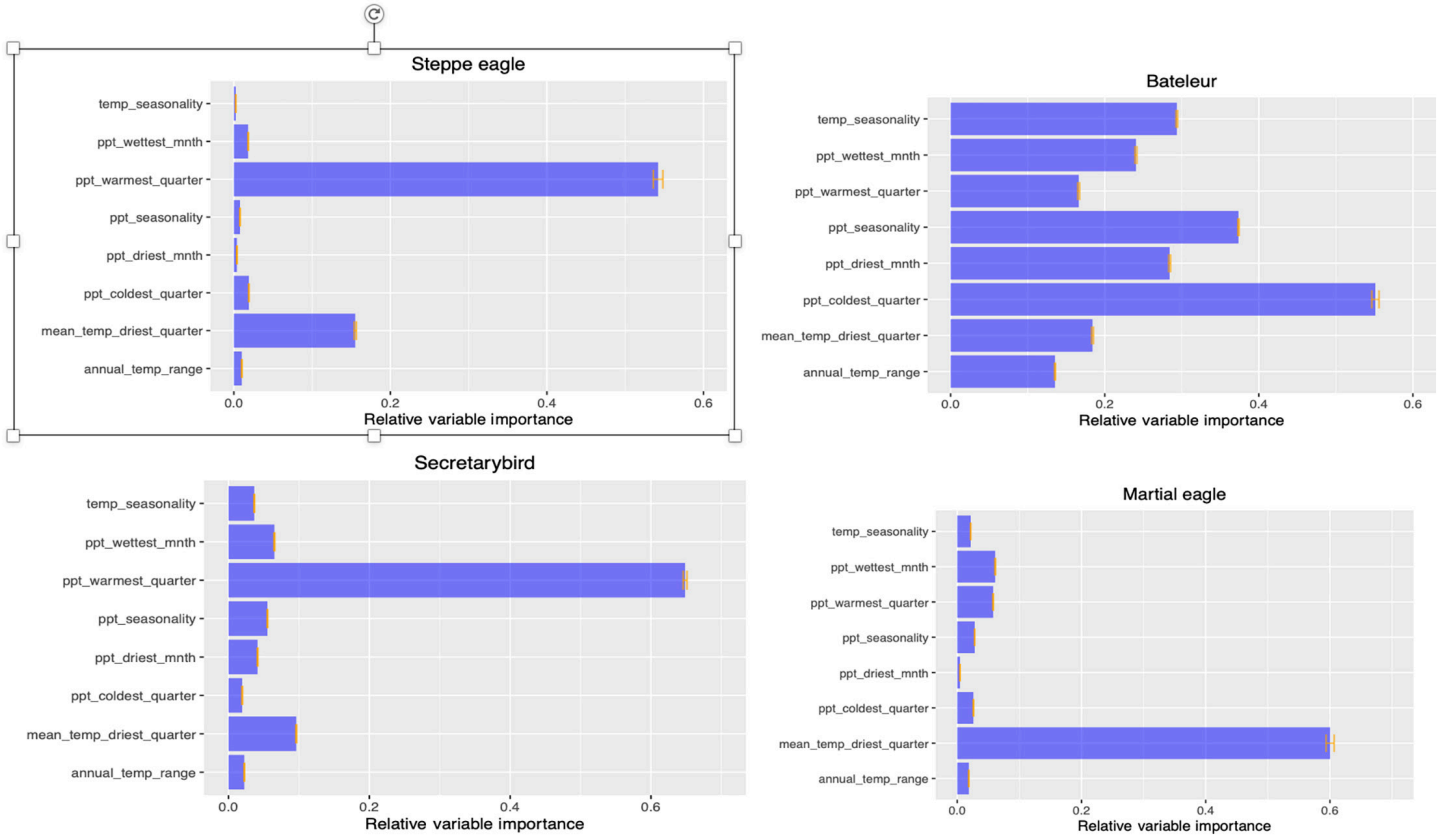
Relative significance of environmental predictor variables to the distribution of niches for raptor species in Kenya. Based on the AUC values of the contributing models, calculated from the ensemble model. The relative importance of the predictor variables ranges from 0 to 1.

The variable importance for all species was computed using the ensemble model, determining the significant factors in predicting their distribution. The ensemble model output revealed variations in the most crucial factors among all species. Nevertheless, similarities were observed between the Secretarybird and Steppe eagle, as both species were influenced by precipitation during the warmest quarter (bio18). In contrast, the distribution of Bateleur was primarily influenced by precipitation during the coldest quarter (bio19) and precipitation seasonality (bio15), according to the model. The distribution of Martial eagles, on the other hand, was primarily influenced by the mean temperature of the driest quarter (bio9) (Figure 4).

### 3.4 Future distributions

**FIGURE 5:**
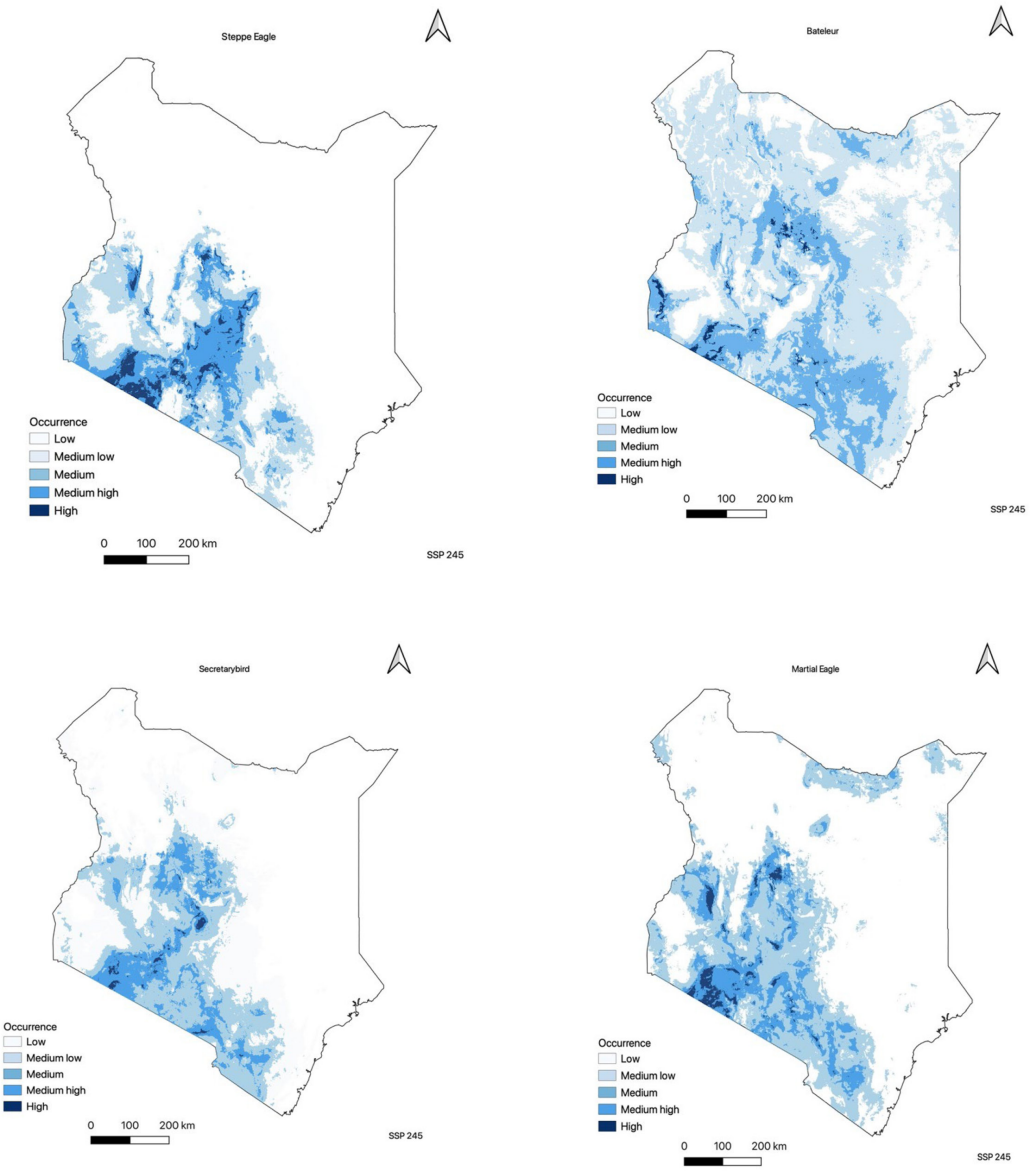
Predicted distribution model of all the four raptor species using future projections (middle of the road scenario). The maps show a continuous logistic prediction with dark blue areas having the highest climatic suitability. White areas show areas with low climatic suitability from the ensemble model.

**FIGURE 6:**
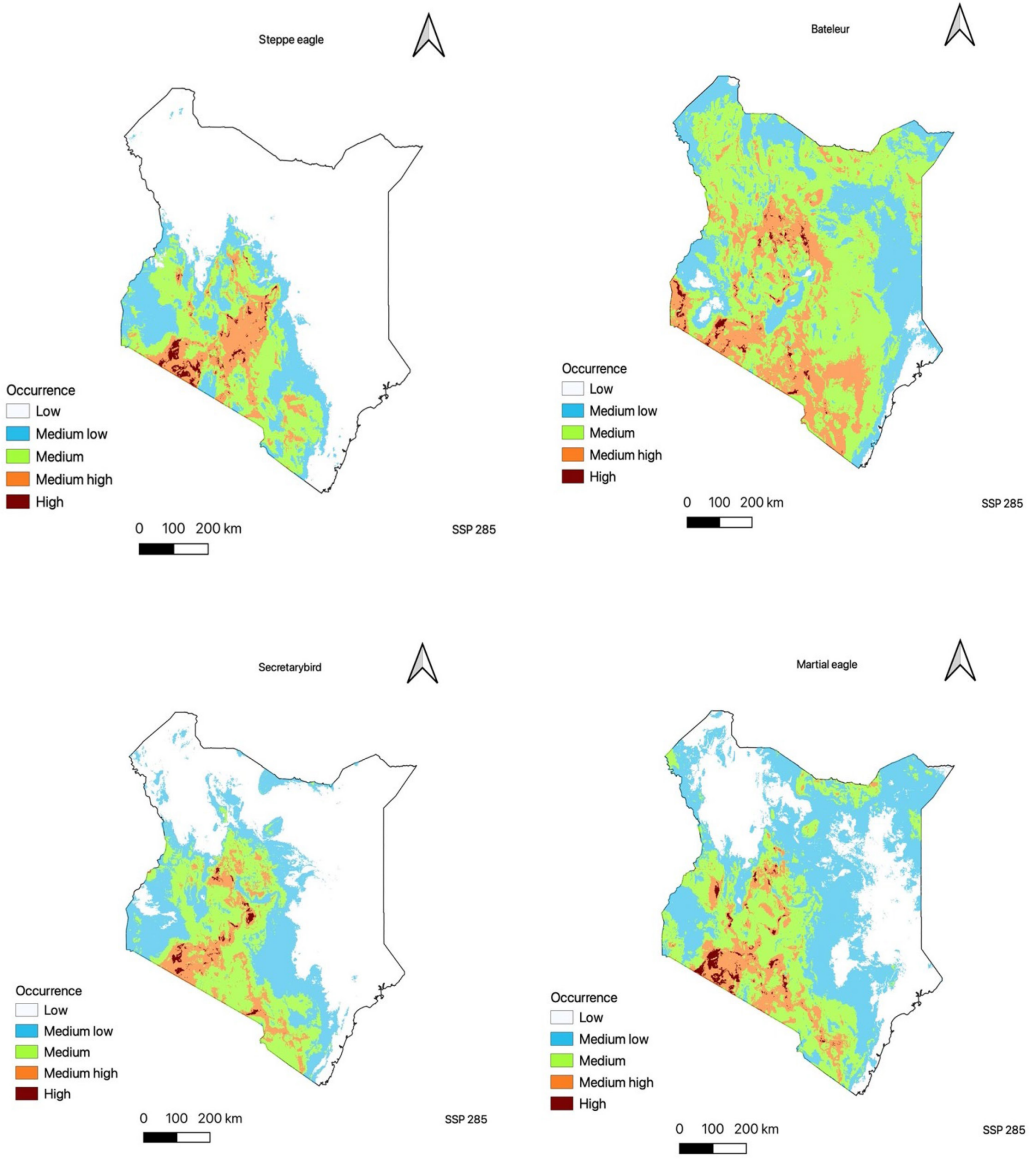
Predicted distribution model of all the four raptor species using future projections (fossil fuel development scenario). The maps show a continuous logistic prediction with maroon areas having the highest climatic suitability. White areas show areas with low climatic suitability from the ensemble model.

The predicted future distributions of the four raptor species as shown in Table 2, Figures 5 and 6 exhibited spatial variations, highlighting the potential existence of suitable climate space for each species in 2040. The analysis revealed considerable diversity in the estimated gain and loss of future suitable climate space across the species (see Appendices 1 and 2 in the additional information section). Across both SSP scenarios, all species displayed a general negative range shift, indicating a potential contraction of their ranges. However, the highest climate suitability was observed in the southwestern part of the country, with some species showing gains in the southern lower region. Notably, among the four species, the Bateleur exhibited a positive gain in the fossil fuel development scenario, with a significant range expansion of +5.601% (247,037 km^2^) from its current range of 233,935 km^2^. Although the Secretarybird experienced a negative change in its species range, the scenario SSP 585 demonstrated a more favorable outcome, with a mean gain of +10.987% (30,062 km^2^), compared to scenario 245, which had a mean gain of +2.729% (7468 km^2^). The Steppe eagle and Martial eagle species range contracted in the future in both climatic scenarios.

**TABLE 2:** Change in suitable climate space for the four raptor species using SSP245 (middle of the road) and SSP 585 (fossil fuel development) climate change scenarios from the HadGEM3 Global circulation model (GCM).

Species	Scenario	Future area (km^2^)	Gain (km^2^)	Gain %	Loss (km^2^)	Loss%	Species change range
Steppe eagle	SSP245	83,108	3871	2.321	−87,524	−52.485%	−50.163
	SSP585	79,823	3632	2.178	−90,570	−54.311%	−52.133
Martial eagle	SSP245	203,828	7468	2.729	**−**77,258	−28.236%	−25.506
	SSP585	179,194	6431	2.35	**−**100,855	−36.86%	−34.509
Secretarybird	SSP245	203,828	7468	2.729	−77,258	−28.236%	−23.627
	SSP585	221,870	30,062	10.987	**−**81,810	−29.899%	−19.065
Bateleur	SSP245	170,430	27,292	11.666	90,797	−38.813	−27.146
	SSP585	247,037	74,835	31.99	61,733	−26.389	5.601

## 4 DISCUSSION

### 4.1 Spatial distribution of raptors and conservation planning

The primary objective of this study was to determine suitable habitats for four endangered raptor species in Kenya based on climatic suitability. Understanding a species' geographic range is crucial in the field of biogeography (Brown et al., 1996) and species distribution models (SDMs) have proven to be valuable tools in spatial conservation planning (Guisan et al., 2013; Lawler et al., 2011) offering substantial potential for meaningful contributions. The priority areas, identified as pivotal habitats for the four species based on our predictive distribution models, were predominantly located in the southern, southwestern, and central regions of Kenya. This distribution pattern is likely influenced by the diverse range of environmental conditions found within the country. For instance, the northern and eastern parts of Kenya experience high temperatures, reaching up to 33°C, and receive minimal rainfall, with <100 mm per season (World Bank, 2022). In contrast, the south‐central and southwestern regions exhibit slightly lower temperatures, with highs reaching 25°C, and receive comparatively higher overall precipitation, exceeding 200 mm in each season (World Bank, 2022).

The climatic suitability for all raptor species tends to be highest in the central regions of their respective ranges, gradually decreasing towards the range edges. These findings highlight the priority areas that require focused conservation management efforts. The models used in this study exhibited strong predictive performance, as evidenced by the evaluation metrics, providing valuable insights into the climatic limitations that shape the distribution of the four raptor species across Kenya. The projected future distributions under the two climate change scenarios revealed diverse range changes in suitable climatic space. Notably, the Bateleur species exhibited a significant expansion of 32% in a lower carbon emission scenario. The species distribution models presented in this study accurately predict the current areas of highest climatic suitability and provide estimates for future suitability. These findings offer crucial guidance for prioritizing conservation actions in the identified regions.

### 4.2 Relative importance of environmental covariates

Understanding the climatic variables that influence species distributions is crucial for designing effective management plans that can adapt to present and future scenarios (Prato, 2012). In our study, the response curves and predictor variable graphs (Figure 4) of our models clearly identify the key climatic characteristics that define the distribution patterns of the four raptor species. Interestingly, both the Steppe eagle and Secretarybird appear to be influenced by similar climatic variables. The distribution of these two species is favored by higher precipitation during the warmest quarter, indicating that increased precipitation corresponds to greater climatic suitability. However, they are also constrained by extremely high temperatures, as indicated by the response curves for temperature during the driest quarter. As temperatures rise, their suitability declines, suggesting a link between optimal seasonal temperature and precipitation for these two species.

In contrast, the Bateleur species demonstrates a higher tolerance for elevated mean temperatures, even exceeding 30°C during the driest quarter, as well as tolerating decreases to an average of 10°C during that same period (bio9). The species appears to be influenced by precipitation levels during the coldest quarter, with higher rainfall acting as a constraint on its distribution (bio19). Conversely, precipitation during the warmest quarter (bio18) appears to favor the distribution of the Bateleur species. The climatic suitability of the Martial eagle species decreased with higher temperatures, as indicated by the response curve for mean temperature during the driest quarter. Interestingly, the species does not appear to be significantly affected by changes in precipitation as its distribution remains constant with various levels of precipitation. Given that precipitation and temperature were the most significant predictors for all four species, the ramifications of changing climate are likely to have a significant impact on how raptors are distributed and may cause their ranges to contract (Phipps et al., 2017).

The resilience of species to climate change is often related to the extent of their geographic ranges (Ofori et al., 2017; Thoya et al., 2022). Given the limited protection currently afforded to raptor priority areas, conservation efforts should extend beyond existing conservation areas. Preserving raptors and the ecosystem services they provide will likely require comprehensive conservation measures and governmental interventions. Legislative actions, such as controlling the distribution and use of veterinary drugs that harm vultures in Africa (Ogada, 2014) may be necessary. Additionally, conducting thorough environmental impact assessments (EIAs) before developing energy infrastructure will help identify and mitigate risks to raptors, particularly vultures (Santangeli et al., 2019). The biodiversity in Kenya's key biodiversity areas (KBAs) faces significant threats and challenges, such as infrastructure development, land‐use changes, illegal activities like logging, and the need for stronger conservation efforts (Barasa et al., 2017). Addressing regional issues like human‐wildlife conflict will be crucial in the decline of raptors.

To improve raptor conservation in Kenya, it is essential to conduct a comprehensive study on the spatial distributions of threats. For instance, studies in Sudan have shown that raptors are susceptible to electrocution from power lines (Angelov et al., 2013) and similar alarming levels of electrocution have been recorded in Kenya (Smallie & Virani, 2010). While increasing renewable energy is important for global environmental sustainability and development in Kenya, it is crucial to minimize the negative impacts of high‐voltage transmission lines on flying birds, including raptors, by implementing appropriate mitigation measures (Barrios & Rodríguez, 2004; Ngila et al., 2023; Sánchez‐Zapata et al., 2016).

### 4.3 Future distributions

The global impact of climate change is expected to cause distributional changes in numerous bird species (Huntley et al., 2006). In our study, future distribution models predict negative range shifts for most raptor species, except for the Bateleur, which shows a positive response under low climatic scenarios. Surprisingly, suitable climatic conditions are projected to contract for the majority of raptor species in both scenarios, except for the Bateleur, possibly due to its ability to tolerate high temperatures amidst global warming. These findings indicate that climate change could significantly impact populations of the Steppe eagle, Secretarybird, and Martial eagle, which already face multiple threats. Therefore, using species‐specific species distribution models (SDMs) calibrated with current best practices becomes essential to effectively predict future distributions (Elith & Leathwick, 2009; Hijmans & Graham, 2006). These results highlight the diverse responses that individual species may exhibit in the face of changing climate conditions.

### 4.4 Study limitations

It is important to acknowledge that the niche and habitat suitability of raptors is influenced by various environmental factors beyond climatic variables alone. The species distribution models (SDMs) used in this study focused exclusively on climatic variables and may not fully capture the complex interplay of other crucial factors affecting raptor distributions. These factors include prey availability (Ontiveros et al., 2005), changes in land use (Smeraldo et al., 2020), elevation (Zhang et al., 2019), and anthropogenic influences (Zhang et al., 2019), among others. Incorporating these additional predictors could enhance the accuracy of the models, providing a more comprehensive understanding of raptor distributions. Furthermore, it is worth noting that future predictions for these non‐climatic factors are currently limited, making it challenging to include them in studies that aim to assess both current and future scenarios. Future research efforts should strive to incorporate these important variables when available, to improve the predictive power of the models and better capture the dynamics of raptor populations in response to changing environmental conditions.

Moreover, it is essential to recognize that this study only considered future climatic scenarios from one Global Climate Model (GCM), namely the HadGEM3. It is well‐documented that different climate models and emission scenarios can yield divergent outcomes. Therefore, caution should be exercised when extrapolating the results to broader contexts, and future studies should consider multiple GCMs and emission scenarios to account for the inherent uncertainties associated with climate projections. Lastly, the use of species‐specific maps can lead to an overestimation of the land area that needs to be protected if each species is assigned its own mapped protected areas therefore a more integrated and ecosystem based approach to conservation planning. We therefore implore caution and emphasize the importance of considering the interconnectedness of ecosystems and the conservation needs of multiple species.

### 4.5 Conclusion

In conclusion, our study has provided valuable insights into the climatic suitability and distribution patterns of four endangered raptor species in Kenya. By utilizing species distribution models (SDMs) and considering future climate scenarios, we have identified priority areas for conservation management efforts. Our findings highlight the importance of understanding species‐specific responses to climate change, as different raptor species exhibited varying degrees of range shifts and climatic constraints. The Bateleur, for instance, demonstrated a positive response under low climatic scenarios, while the Steppe eagle, Secretarybird, and Martial eagle were found to be particularly vulnerable to the impacts of climate change. Future research should focus on spatially identifying and understanding the specific threats to raptors in order to develop targeted conservation strategies. Incorporating landscape‐level conservation strategies such as establishment of ecological corridors or connectivity networks can enhance habitat connectivity and facilitate the movement of species across landscapes. This approach can promote the preservation of ecological processes and allow species to adapt and respond to changing environmental conditions, including the impacts of climate change. Overall, our study underscores the need for proactive conservation measures, integrated approach to conservation planning, strong policy interventions, and robust monitoring programs to safeguard raptor populations and maintain the ecological balance in Kenya. By prioritizing the conservation of raptors, we can contribute to the preservation of biodiversity and the long‐term sustainability of the natural environment.

## AUTHOR CONTRIBUTIONS

**Peggy Mutheu Ngila:** Conceptualization (equal); formal analysis (equal); methodology (equal); software (equal); writing – original draft (equal). **David Odhiambo Chiawo:** Conceptualization (equal); funding acquisition (lead); resources (lead); supervision (lead); writing – original draft (equal). **Margaret Awuor Owuor:** Conceptualization (equal); funding acquisition (equal); project administration (equal); writing – original draft (equal). **Vivian Oliver Wasonga:** Conceptualization (supporting); project administration (lead); supervision (equal); writing – original draft (equal). **Jane Wangui Mugo:** Formal analysis (equal); methodology (equal); software (equal).

## CONFLICT OF INTEREST STATEMENT

The researchers acknowledge that they are not aware of any personal or financial conflicts that might have affected the research presented in this document.

## Data Availability

The data that support the findings of this study are available in GBIF at https://doi.org/10.15468/dl.hzyp6v. These data were derived from the following resources available in the public domain: [Repository: Global biodiversity information facility (GBIF)] The data that supports the findings of this study are openly available in Global biodiversity information facility (GBIF). Raptors occurrence data at https://doi.org/10.15468/dl.hzyp6v.
